# Cardiac impact of high-frequency irreversible electroporation using an asymmetrical waveform on liver in vivo

**DOI:** 10.1186/s12872-021-02412-9

**Published:** 2021-12-07

**Authors:** Jing Li, Jingjing Wang, Xiaobo Zhang, Xiao Zhang, Hongmei Gao, Yueyong Xiao

**Affiliations:** 1 Department of Radiology, The First Medical Center of Chinese, PLA General Hospital, Beijing, China; 2 Department of MRI, Affiliated Hospital, Logistics University of Chinese Peoples Armed Police Forces, Tianjin, 300162 China; 3 Department of Critical Care Medicine, Tianjin First Center Hospital, Tianjin, 300192 China

**Keywords:** High-frequency irreversible electroporation, Irreversible electroporation, Liver ablation, Arrhythmia, Cardiac injury

## Abstract

**Background:**

High-Frequency Irreversible Electroporation (H-FIRE) is a novel technology for non-thermal ablation. Different from Irreversible electroporation (IRE), H-FIRE delivers bipolar electrical pulses without muscle contraction and does not cause electrolysis. Currently, little is known regarding the cardiac safety during the administration of H-FIRE on liver. The aim of this study was to evaluate the changes of electrocardiogram (ECG) and biomarkers of cardiac damage during asymmetrical waveform of H-FIRE therapy in vivo.

**Methods:**

The swines (n = 7) in IRE group, which used 100 pulses (2200 V, 100–100 μs configuration), were administrated with muscle relaxant under anesthesia. In the absence of muscle relaxant, 7 swines in H-FIRE group were performed with 2400 pulses (3000 V, 5–3–3–5 μs configuration). Midazolam (0.5 mg/kg) and xylazine hydrochloride (20 mg/kg) were given to induce sedation, followed by Isoflurane (2.5%, 100% oxygen, 3 L/min) to maintain sedation in all the swines. Limb lead ECG recordings were analyzed by two electrophysiologists to judge the arrhythmia. Cardiac and liver tissue was examined by pathology technique.

**Results:**

The ablation zones were larger in H-FIRE than IRE. Both IRE and H-FIRE did not affect the autonomous cardiac rhythm. Even when the electrical signal of IRE and H-FIRE fell on ventricular vulnerable period. Moreover, cTnI in IRE group showed an increase in 4 h after ablation, and decreased to baseline 72 h after ablation. However, cTnI showed no significant change during the administration of H-FIRE.

**Conclusions:**

The study suggests an asymmetrical waveform for H-FIRE is a promising measure for liver ablation. The results were based on normal liver and the swines without potential cardiac diseases. With the limitations of these facts, asymmetrical waveform for H-FIRE of liver tissue seems relatively safe without major cardiac complications. The safety of asymmetrical waveform for H-FIRE needs to evaluate in future.

## Background

Irreversible electroporation (IRE) is a non-thermal ablation technology that creating irreversible nanoscale pores in the cell membrane and leading to cell death through apoptosis [1]. Based on the application of high voltage and short monopolar pulses with two electrodes placed across the target site, IRE has been implemented to ablate primary and metastatic hepatic or pancreatic tumors near thermosensitive structures. IRE was known to cause muscle contractions during treatment. Sedative and neuroparalytic agents should be used in patients undergoing invasive mechanical ventilation [2]. Moreover, IRE adhere to the heart can be cardiac arrhythmia when the electrical pulse delivered outside of the absolute refractory period of the cardiac cycle [3, 4]. Thus, synchronization was recommended to use during the operation of IRE in case of fatal cardiac arrhythmia [5, 6].

High-Frequency Irreversible Electroporation (H-FIRE) is the next-generation nanoknife ablation technology to conquer many of the technical barriers of conventional IRE as mentioned above [7]. Previous studies have showed that H-FIRE, which delivered ultra-short bipolar electrical pulses, minimized the stimulation of nerve and muscle. There is widespread speculation that H-FIRE could be used without ECG monitoring and taking no account of synchronization with R wave on ECG [8]. However, it is well-known that the principle of electroporation is permeabilization of the cell membrane around the electrode. The pore produced by electroporation affected the transportation of ion across the cell membrane. In reality, whether H-FIRE affects the cardiac electrophysiology is still unknown.

There are two wave-forms of H-FIRE. In theory, asymmetric waveform is more effective than symmetrical waveform [9]. Es et al. had demonstrated an asymmetric waveform for H-FIRE creates deeper lesions than symmetric waveform of the same energy and frequency [10]. However, the cardiac safety did not evaluate. The purpose of the present study is to evaluate cardiac safety in vivo with asymmetric waveform of H-FIRE in contrast to the conventional IRE.

## Methods

### Animals

All animal experimental protocols were approved by the Institutional Animal Care and Use Committee of The General Hospital of People’s Liberation Army (License Number: 2019-D15-07). The study was conformed to the National Institutes of Health Guide for Animal Care and Use of Laboratory Animals (NIH Publication No. 85-23, revised 2011). Female Ba-Ma miniature pigs weighed 20–25 kg were used for this study (Shi-chuang Animal Labs, Beijing, China).

### Surgical procedures

Experiments were carried out with midazolam (0.5 mg/kg) and ketamine (10 mg/kg) for induction and Isoflurane inhalation (1–3%) for maintenance. All the swines were under intubation and mechanical ventilation. Animals were continuously monitored using telemetry and pulse oximetry. Of the 14 animals, 7 animals underwent IRE, the remaining 7 animals underwent H-FIRE. Before delivery of the IRE, the swines received vecuronium (0.05–0.1 mg/kg) to limit muscle contraction. All the animals were sacrificed within 72 h of the procedure. An overdose of pentobarbital sodium (150 mg/kg) was used to sacrifice all the animals.

**Fig. 1:**
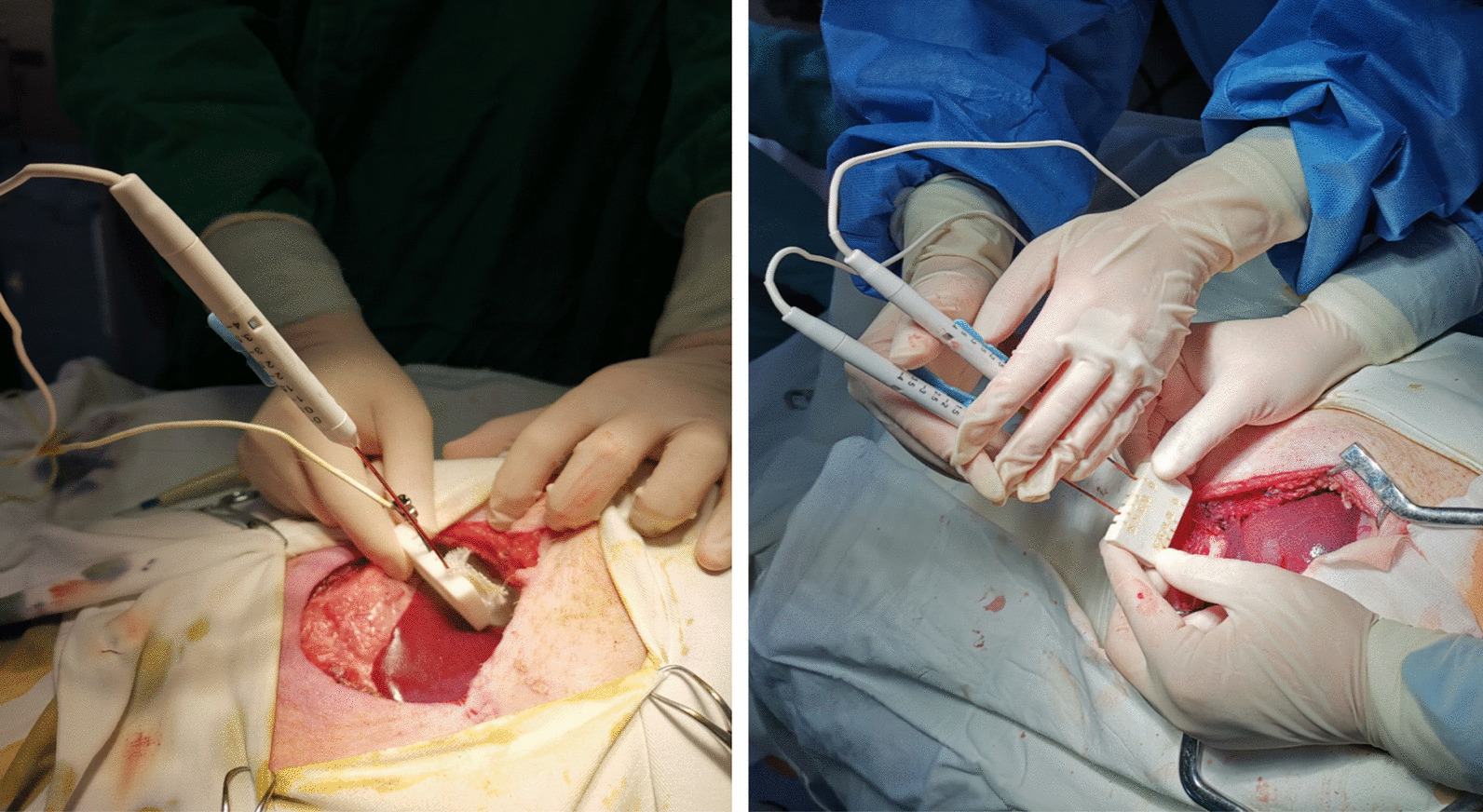
Intra-operative image demonstrating liver placement of electrodes. Placement of IRE delivery device (left). Placement of H-FIRE device (right)

The upper midline incision (15–20 cm) was made and the liver was exposed. Subsequently, the two platinum-iridium electrodes of IRE or H-FIRE were positioned in parallel with spacing set to 1.5 cm on the lateral segment of the liver, and the active portion of the electrodes exposed to a length of 1.5 cm. The electrodes were inserted into the hepatic parenchyma to a final depth of 2.5–3.0 cm (Fig. 1).

### IRE and H-FIRE protocol

**Fig. 2:**
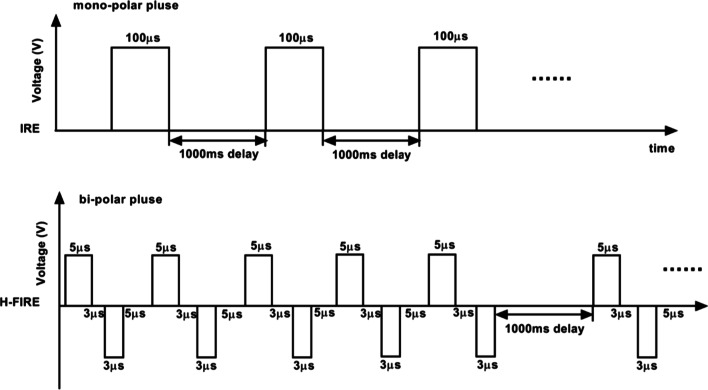
Schematic representation of electrical pulse delivery using IRE (upper) and H-FIRE (lower)

IRE (YTL-GM01, Inteligent Health, Tianjin) delivery was using computerized algorithms and square pulses 2200 V was delivered without R wave synchronization. Each IRE treatment group consisted of 10 pulses (total 10 groups and 100 pulses), each pulse sustained 100 microseconds with a 1000 ms pause between each group of pulses (Fig. 2, Table 1).

**Table 1:** The parameters of IRE and H-FIRE ablation

	IRE	H-FIRE
Number of pulses	100	2400
Voltage	2200 V	3000 V
Pulse width	100 μs	5 μs (postive) and 3 μs (negative)
Pulse direction	Unidirectional	Bidirectional
Pulse interval	1000 ms	1000 ms
Number of pulses in one group	10	10
Number of group	10	240
Positive and negative pulse interval	None	3 μs

H-FIRE was generated by a custom-built pulse generation system (Inteligent Health, Tianjin) with the ability to deliver microsecond, biopolar pulses in rapid bursts. The positive voltage presents pulse width of 5 μs, with a delay of 3 μs between each change in polarity. The following negative voltage presents pulse width of 3 μs, with a delay of 5 μs between each change in polarity (Fig. 2). A total of 2400 bursts at a 3000 V amplitude was set per sequence, and with a 1000 ms pause between each group of pulses (Table 1).

### Cardiac monitoring

Cardiac rhythms was recorded for each procedure with a single-lead ECG (XD-7100, Tai yi, Shang hai, China). Animals were divided into two groups underwent 100 or 2400 bursts of liver ablations using IRE or H-FIRE, respectively. These ECG rhythms were analyzed by two electrophysiologists back to back. An ECG was recorded every 5 min for up to 30 min after ablation. Major cardiac complications are defined as ventricular fibrillation, ventricular tachycardia, high degree Atrio-Ventricular blocks (AVB).

Two venous access were obtained on each sides of the groins using 8F introducers. The biomarker of cardiac injury were obtained from the blood samples before IRE or H-FIRE intervention, 4 h and 72 h after IRE or H-FIRE intervention. Cardiac Troponin I (cTnI, normal range 0–1.68 ng/L, cTnI assay on Dimension Vista 1500 system, Siemens Healthineers, Erlangen, Germany) was detected.

IRE-treated, H-FIRE treated heart were fixed in formalin, and sliced for haematoxylin and eosin (H&E) staining. The diagnoses of hearts were made by two senior attending doctors blinded in the pathology department.

### Magnetic resonance imaging (MRI) imaging and analysis

All images were acquired using an abdominal coil (32-Channel abdominal Coil; GE Medical, Fairfield, USA) in a 3.0-T clinical MRI unit (GE, MAGNETOM 750 W 3.0 T), with the animal in a prone or lateral position. Similar to prior studies [10], the ablation zone size was defined as the unenhanced region on contrast-enhanced MRI with reduced signal intensity as compared with normal liver tissue. Determine whether an effective ablation was performed.

### Histology

Sections were cut at a thickness of 5 µm, and representative sections were stained with hematoxylin & eosin (H&E). The diagnoses were made by a senior attending doctor in the Pathology Department.

### Statistical analysis

Statistical analysis was performed using GraphPad Prism version 8 software (GraphPad Prism Software Inc., San Diego, CA, USA). Continuous variables are expressed as mean ± standard deviation (SD). Categorical variables were summarized as numbers (%). The abnormal changes of ECG between IRE group and H-FIRE group was compared using Fisher's exact tests. The change of cardiac biomarkers between IRE group and H-FIRE group was compared using t-tests. A *P* value < 0.05 was considered significant.

## Results

### IRE and asymmetrical waveform of H-FIRE performed effective ablation in this study

**Fig. 3:**
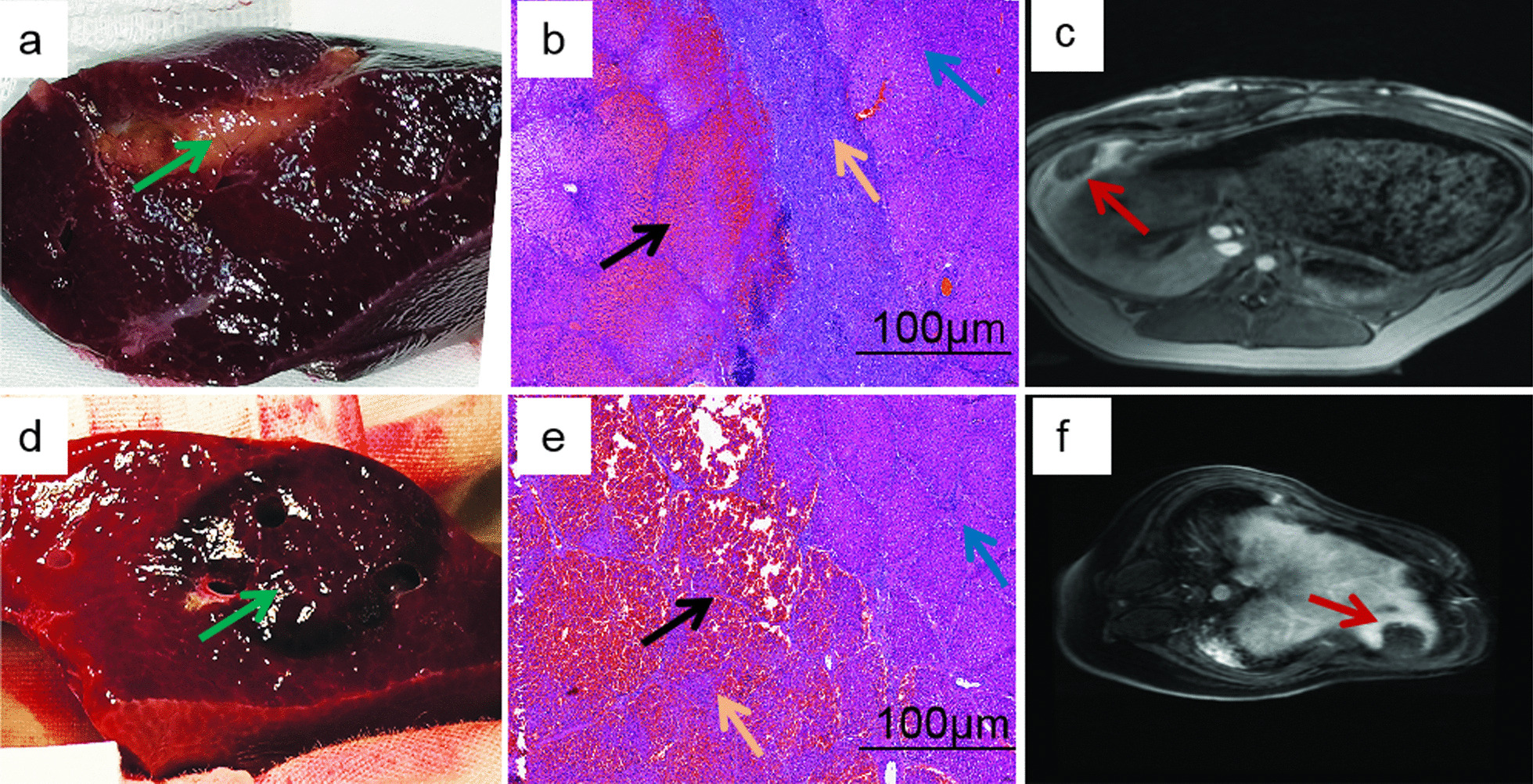
Comparison of hepatic ablations using H-FIRE with IRE. **a** and **d** Representative gross pathology images illustrating sections of ablation zones for 72-h after IRE and H-FIRE ablation, respectively. **b** and **e** Pathological images (H&E staining) for 72-h after IRE and H-FIRE ablation, respectively. **c** and **f** MRI images in coronal orientation for 72-h after IRE and H-FIRE ablation, respectively. Represents ablation zone in gross specimen of liver. Represents large areas of hemorrhagic infiltration. Represents normal liver tissue. Represents the infiltration of inflammatory cells. Represents region of interests (ROIs) of the ablation zone on contrast-enhanced MRI

Gross tissue revealed an oval shaped ablation zone. Long axis of ablation zone was parallel to the emission electrodes (Fig. 3a, d). Following HE staining, the ablation area showed the normal liver structure was damaged, and instead of large areas of haemorrhage as well as inflammatory cell infiltration (Fig. 3b, e). Ablative lesions in liver tissue was evaluated with contrast-enhanced MRI. All lesions appeared as a focal or ovoid area. Ablation zone on contrast-enhanced MRI was with reduced signal intensity as compared with normal liver tissue (Fig. 3c, f).

### The effect of the depolarization and repolarization of ventricle was similar

**Fig. 4:**
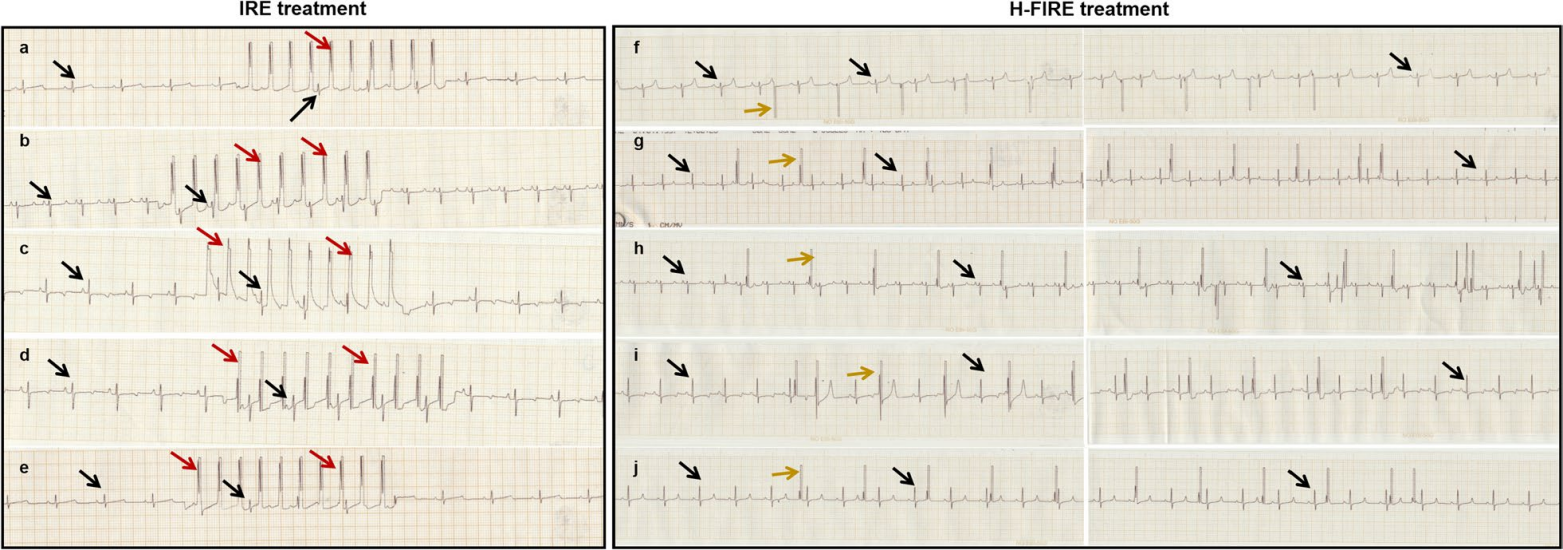
ECG of five piglets in each group with the change of QRS waveform in the ablation procedure. **a**–**e** ECG of five piglets during IRE ablation. **f**–**j** ECG of five piglets during H-FIRE ablation. Represents autonomous cardiac rhythm. Represents electrical signal of IRE. Represents electrical signal of H-FIRE

Of the fourteen piglets, seven received IRE and the remaining seven received asymmetric waveform of H-FIRE. No matter where the electronic signal occurred, there was no significant changes in QRS waveform and QRS duration of the original sinus excitation. In this animal study, we did not notice a significant drop or elevation in myocardial voltage after IRE or H-FIRE. None of the piglets developed ST-segment deviations or T-wave changes related to the ablation of IRE or H-FIRE (Fig. 4). The current of each ablation are summarized in Table 2.

**Table 2:** The current in IRE and H-FIRE ablation

IRE (A)	H-FIRE (A)
14–16	14–16
15–18	10–12
10–12	10–12
11–15	10–14
12–13	8–12
12–14	11–13
13–15	12–14

In IRE nad H-FIRE, the current fluctuates in a range. this table is reflect the range of current

### The histology of heart accepted IRE and H-FIRE treatment were normal

**Fig. 5:**
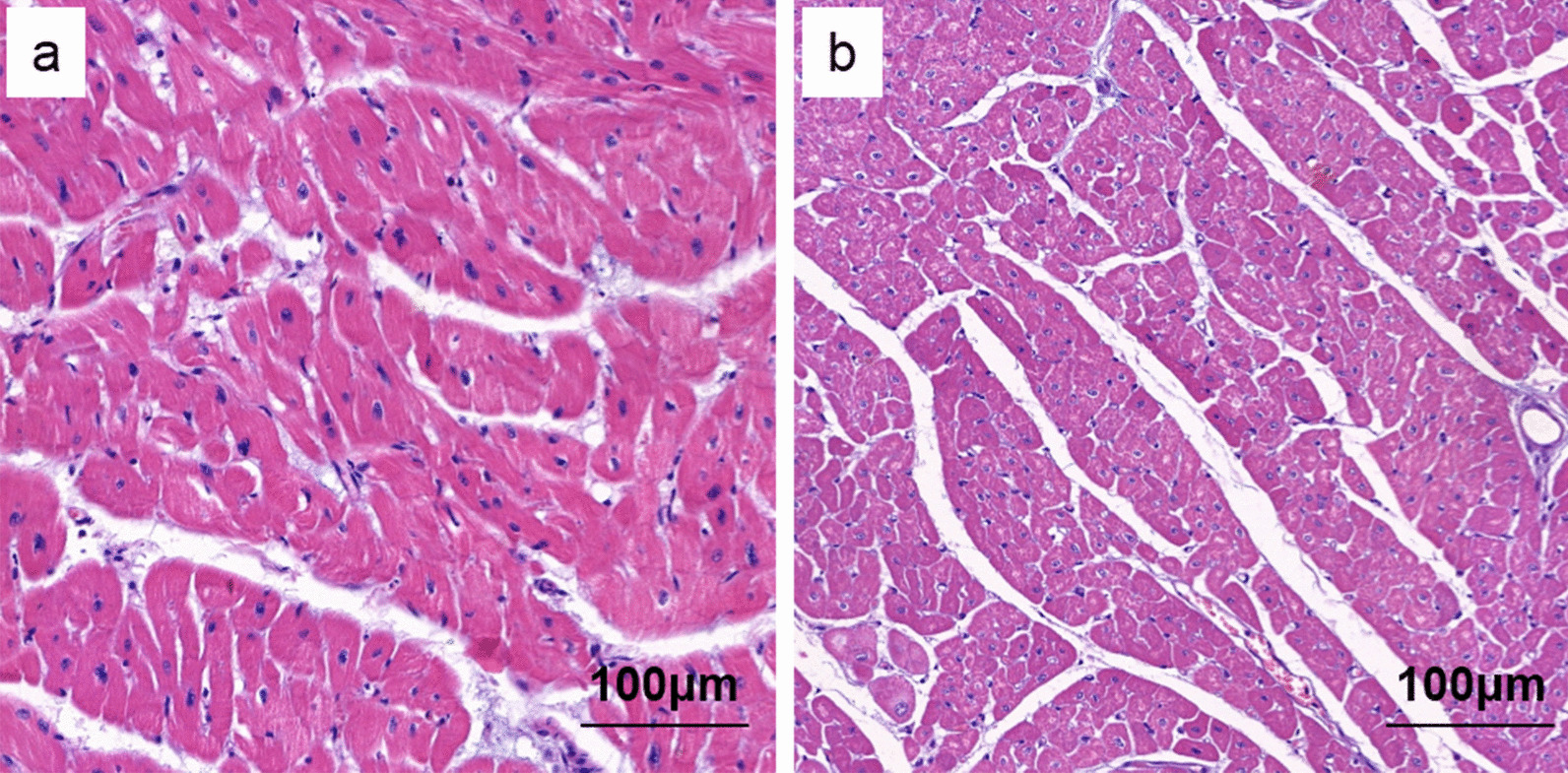
Comparison the histology of heart with IRE and H-FIRE treated. **a** The histology of heart with IRE. **b** The histology of heart with H-FIRE. Both a and b was normal without myocardial injury

Both hearts tissue with IRE (7/7) and H-FIRE (7/7) treated were normal. There was no injure of heart can be seen (Fig. 5).

### Cardiac biomarkers demostrated the safety of H-FIRE

**Fig. 6:**
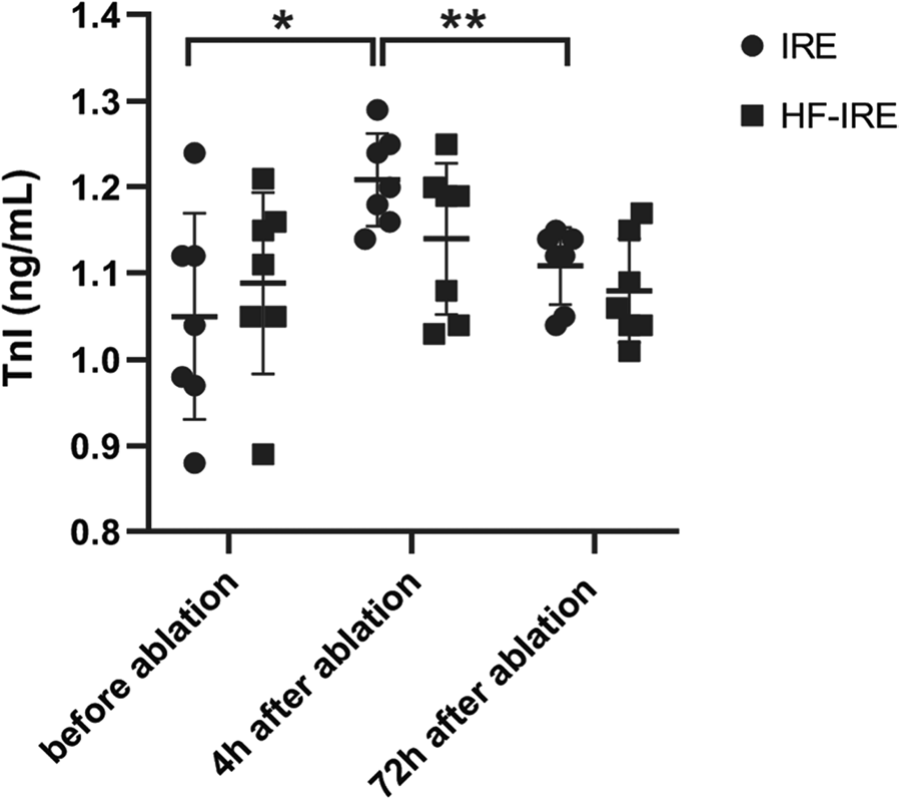
Comparison of TnI level using H-FIRE with IRE. The concentration of cTnI after 4 h of IRE ablation increased, then cTnI level decreased to baseline after 72 h of IRE ablation. cTnI level before and after H-FIRE ablation did not alter. *P < 0.05; **P < 0.01. n = 7

Regarding cardiac biomarkers, IRE group showed an increase in cTnI after four hours of the ablation. However, the concentration of cTnI after 72 h of the ablation was lower to baseline. Whereas, the level of cTnI showed no significant change in H-FIRE group during the ablation (Fig. 6).

### None minor or major events of IRE and H-FIRE occurred

All animals survived and tolerated IRE or H-FIRE delivery until sacrifice. None of the 14 animals experienced a minor or major arrhythmic complication.

## Discussion

In the present study, we evaluated the cardiac safety of a novel asymmetrical waveform for H-FIRE, and compared it to IRE of the equivalent energy. In this study, the ablation efficacy of H-FIRE was similar to IRE of liver. IRE group showed an increase in cTnI after four hours of the ablation. However, the concentration of cTnI after 72 h of the ablation was lower to baseline. Whereas, the level of cTnI showed no significant change in H-FIRE group during the ablation. In consistent with previous study, these results were due to the maximum current and the intensity of energy release applied of IRE was higher than H-FIRE [11].

Cardiac depolarization and repolarization are depended on ion flux alteration inside and outside of the cardiac cells. External electrical stimuli can alter cell membrane potential, leading to a localized depolarization [12]. The fundamental of electroporation is permeabilization of the cell, which can induce cardiac arrhythmia [3]. Previous studies suggested that the current electricity may trigger cardiac arrhythmia during and after IRE ablation near to the heart. Therefore, IRE was modified delivering electric impulses with ECG synchronization [3, 4]. Different from IRE, H-FIRE delivers bipolar pulses of 1–5 μs in a rapid burst with the benefits in reducing nerve and muscle stimulation [7]. These properties allow the administration of H-FIRE without the requirement of paralytics. Theoretically, it is speculated that H-FIRE obviates the requirement of cardiac synchronization. There are two waveform for H-FIRE. Moreover, asymmetric H-FIRE is more effective than symmetrical H-FIRE in vivo [10]. Previous report showed that no EKG abnormalities during symmetrical H-FIRE ablating the hepatic tissue in swines [13]. Thus, specific safety issues on the heart with asymmetric H-FIRE should be discussed. It is well known that distance is key to avoid cardiac arrhythmia. Previous study of IRE suggested that synchronization may be necessary at distances greater than 1.7 cm from the myocardium (as measured on an axial CT scan) [5]. We speculated that the results in our study might due to the electric pulses on liver was a stimulus of remote field.

It is worthy that the effective refractory period was a fundamental prerequisite for the maintenance of fibrillation conduction. If electrical stimuli fall on the vulnerable period, it can induce cardiac malignant arrhythmia. In contrast, if electrical stimuli fall during the absolute refractory period, arrhythmogenic potential is minimized. Refractory period of the ventricular myocardium refers to the starting of QRS complex to the peak of T wave. Vulnerable period of the ventricular myocardium refers to the peak of T wave to the ending of T wave (Tpeak–Tend). In our study, most of the electrical stimuli detected in ECG arises on QRS complex of the swine’s autonomous cardiac rhythm. Although H-FIRE pulse was arised on the vulnerable period, no existing dysrhythmia was seen of H-FIRE. ECG is the tracing of cardiac electrical activity. Electrical pulses of H-FIRE detected on ECG did not mean the stimuli conducted to the cardiac cell. It is the limitation of our study that the intra-cardiac electrical activity did not examine. We did not know whether the electrical signal of H-FIRE delivered on vulnerable period could cause cardiac arrhythmia. QT interval, QTc interval, and Tpeak–Tend interval are markers of cardiac arrhythmia. Both prolonged QT and QTc [14] and shortened QT interval and QTc [15] are associated with malignant ventricular arrhythmia (VAs). Prolonged Tpeak–Tend is associated with an elevated arrhythmic risk [16]. Compared with the ECG before H-FIRE ablation, QT interval, QTc and Tpeak-Tend did not change in the present study. These results suggested that no electrocardiogram abnormalities were observed during H-FIRE for asymmetrical waveform. Whereas, our results were contrast to a meta-analysis, which showed that the incidence of cardiac arrhythmias related to IRE on liver was 16% (21 of 129 patients) [17]. Due to the patient with cardiac comorbidity was prone to cardiac arrhythmia. In addition, we found the piglets had an increase in cTnI after 4 h of IRE ablation. However, the concentration of cTnI decreased to baseline after 72 h of IRE ablation, indicating cardiac injury is temporary. Moreover, level of cTnI had no significant change during the administration of H-FIRE. That suggested H-FIRE maybe safe than conventional IRE. Symmetric H-FIRE is safer than IRE and asymmetric H-FIRE for the pluses cancelled each other out. But the ablation efficiency also could be impacted. Asymmetric H-FIRE is the balance between safe and efficiency.

Our study has many limitations. First, the number of animals used for this study was small. Second, whether stimulation on liver with asymmetrical H-FIRE has an effect on the heart, future studies on H-FIRE should incorporate measurements of intra-cardiac potential. Third, this study was based on normal porcine model, future studies about cardiac safety of H-FIRE needs to evaluate in patients with liver tumor.

## Conclusions

In conclusion, these results showed H-FIRE with asymmetrical waveform seems relatively safe without major cardiac complications on the normal liver without tumors and the piglets without any potential cardiac diseases.

## Data Availability

The datasets used and/or analyzed during the current study available from the corresponding author on reasonable request.

## Abbreviations

H-FIRE: High-Frequency Irreversible Electroporation
IRE: Irreversible electroporation
ECG: Electrocardiogram
cTnI: Cardiac Troponin I
ROI: Region of interests
